# Isolation and characterization of Leucine dehydrogenase from a thermophilic *Citrobacter freundii* JK-91strain Isolated from Jask Port

**Published:** 2013-09

**Authors:** Rahman Mahdizadehdehosta, Anvarsadat Kianmehr, Ahmad Khalili

**Affiliations:** 1 Department of Biology, Islamic Azad University, Bandar Jask Branch, Bandar Jask, Iran; 2 Department of Medical Biotechnology, School of Advanced Medical Sciences, TabrizUniversity of Medical Sciences, Tabriz, Iran; 3 Department of Immunology, Tarbiat Modares University, Tehran, Iran

**Keywords:** Characterization, *Citrobacter freundii*, Leucine dehydrogenase (LeuDH), JaskPort, Thermophile

## Abstract

**Background and objectives:**

Leucine dehydrogenase (LeuDH; EC 1.4.1.9) belongs to the amino acid dehydrogenase family and isused as a biocatalyst in medical and pharmaceutical industries (1). This study reported deals with the isolation and characterization of LeuDH from a thermophilic bacterium isolated from Jask Port in the Province of Hormozgan.

**Materials and Methods:**

Aliquots of soil and water samples were cultured in LEU specific medium and thermophilc bacteria that exhibited LeuDH activity were isolated and characterized biochemically. The LeuDH was purified and characterized in regard to the effects of pH and temperature on the activity, as well as its molecular weight determination.

**Results:**

A thermophilic bacterium, *Citrobacter freundii* strain JK-9 was identified and found to exhibit LeuDH activity. The enzyme characterization revealed that LeuDH exhibits higher activity at temperature range of 60 to 75°C (optimum of 60°C) and an optimum pH of activity at pH 10.5. The *K*_m_ value of LeuDH is 1.2 mM, while its molecular weight is about 320 kDa, and consisted of eight subunits identical in molecular mass (40 kDa).

**Conclusion:**

Briefly, a thermostableLeuDH enzyme from a strain of *C. freundii* was isolated and characterized. Our data indicate that the *C. freundii* enzyme has potential for use in biotechnological applications.

## INTRODUCTION

The enzyme, Leucine dehydrogenase (LeuDH; NAD^+^oxidoreductase, deaminating; EC 1.4.1.9)belongs to the amino acid dehydrogenase family of enzymes which catalyzes the reversible oxidative deamination reaction of L-leucine and other branched chain amino acids to their respective α-ketoacids (1). An assay technique with a high sensitivity for blood L-Leucine level, an important marker for the screening of Maple Syrup Urine Disease (MSUD), has been established by means of LeuDH (2). This enzyme is being used as a commercial and valuable biocatalyst in medical and pharmaceutical industries (1, 2). LeuDH has been applied to measurement of blood L-leucine in diagnostic kits for the neonatal screening of MSUD (3, 4). Mental retardation, seizure, auditory failure, strabismus, and kidney failure are the complications of MSUD. Timely diagnosis and prevention are the most efficient strategies for control and management of MSUD (5, 6). LeuDH plays a role in synthesis of branched chain amino acids as well as L-tert-leucine, a building block of antibiotics. Quantification of leucine amino peptidase, a liver disease marker is another application of the mention edenzyme (1). With respect to the LeuDH applications, finding enzyme with special features like temperature resistance or reaction in high saline concentration scan be very interesting. Thermophilic enzymes are known to not only tolerate high temperatures but also retain their activities in the presence of organic solutions, acidic and basic mediums and detergents (1). LeuDH has been isolated and characterized from different bacteria species including *Bacillus cereus, B. sphaericus, B. subtilis, Natorobacteriummagadi* (7–9). Among the thermophilic bacteria producing the target enzyme, it can be referred to *B. sphaericus, Thermoactinomyces intemediuc*, and *Clostridium thermoaceticus* (10). The search for extermophilic organisms is one of the means for finding enzymes with features suitable for industrial applications. The main advantage of thermostable enzymes is that they are able to tolerate higher temperature, which gives a longer half-life to the enzyme (1). Although, many studies related to isolation of theromstable LeuDH producing microorganisms have been carried out in the world, however, no such work has been done in Iran so far. In this research with the aim of isolation of LeuDH enzyme with considerable characteristic like thermophilicity, screening from collected samples of water and soil microorganisms from the Jask Port in Provinceof Hormozgan was implemented.

## MATERIALS AND METHODS

NAD^+^ and NADH were purchased from Sigma-Aldrich Corp (USA). All amino acids including L-Leucine, L-Isoleucine, L-Valine, L-Alanine, D- Leucine, L-Arginine, Aspartate, Glycine, and L-Threonine were prepared from Merck (Germany). All other chemicals such KH_2_PO_4_, NaCl, and Tris-HCl were from Sigma-Aldrich. 10 different water and soil samples were collected from various regions of the Jask Port in Province of Hormozgan. Soil and water samples were maintained in sterilized bottles and freezer bags, respectively.

### Microorganisms and culture conditions

In order to isolate microorganisms that are capable to metabolize L-leucine, firstly one gram of each soil sample and 1 ml of each water sample were taken and added separately to LEU medium [1% L-Leucine (w/v), 0.2% KH_2_PO_4_ (w/v), 0.2% NaCl (w/v), 0.01% MgSO_4_ (w/v), 0.01% yeast extract, pH7.2] and then incubated at 60°C and 130 rpm for 48 h. From each culture, aliquot was transferred to agar plate and incubated at 60°C for 48 h. Grown colonies were isolated based on their morphological features and transferred to same new medium (11).

### Screening for LeuDH producing bacteria

In order to screen and identify LeuDH producing strains, the isolated thermophilic bacteria were grownin LEU medium at 60°C for 48 h under aerobic conditions. The cells were harvested by centrifugation, washed once with 0.9% NaCl solution and resuspended in10mM potassium phospahte buffer (pH 7.2) containing 0.1 mM EDTA and 5mM 2-mecaptoethanol and then disrupted by 9-KHz ultrasonic oscillator for 20 min. The cell debris was removed by centrifugation and supernatant solution was used for enzyme screening. LeuDH activity was assayed by measuring NADH formation at 340 nm spectrophotometricaly (Shimadzu UV-visible-1601 PC, Japan). The reaction mixture constituted: 100 mML-leucine, 50 mM glycine-NaOH buffer (pH 10.4), 100 mM NaCl, 2.5 mM NAD^+^ and enzyme solution in a total volume of 1.0 ml. One unit of enzyme activity was defined as the amount of enzyme that produced 1 µmol of NADH per min in the oxidative deamination of each amino acid. Also the specific activity was expressed as units per milligram of protein. The total protein concentration was determined using a Bio-Rad protein assay kit with bovine serum albumin (BSA) as the standard (11).

### Strain identification

Identification of bacterium was performed by morphological characterization, biochemical methods and specific PCR amplification. Classification as Gram negative or Gram positive was done by Gram stain reaction. Morphological characteristics of the isolated bacterium were also performed according to the standard method (12). Biochemical methods were conducted using well-established biochemical tests described in Bergey's manual of Determinative Bacteriology (13). Chromosomal DNA from bacterial cell was purified according to the Doi protocol (11). The resultant DNA sample was suspended in a buffer containing 10 mM Tris-HCl (pH 7.6), 1 mM EDTA and 10 mM NaCl and stored at -20 °C for further work. Complete 16S rRNA gene sequence was amplified with primers 16SF (5'- TGCAAGTCGAACGGTAGCA -'3) and 16SR (5'- CGTTGCATCGAATTAAAC -'3). PCR amplification was performed in volumes of 25 μl containing 20 pmol of each primer, 1X PCR buffer, 0.2 mM of each dNTP, 1.5 mM MgCl_2_, 0.4μg template DNA and 2.5 units of *Taq* DNA polymerase. The resultant PCR product was then analyzed in a 1.5% (w/v) horizontal agrose gel. Sequencing was performed by the commercial services of MacroGen Co. ltd. (Seoul, Korea) with the appropriate sequencing primers. The 16S rDNA sequence of the isolate was aligned with the reference 16S rDNA sequences using the Basic Local Alignment Search Tool (BLAST) algorithm available in NCBI (National Center for Biotechnology Information) in internet. Multiple alignment of sequences and calculations of levels of sequence similarity were performed by using Clustal W. Phylogenic trees were constructed via the neighbor-Joining (NJ) algorithm using the Molecular Evolution Genetic Analysis (MEGA) program, version 5.0 (14). Bootstrap test was done 1000 times to confirm the reliability and validity of inferred trees.

### Enzyme Purification

Purification of LeuDH was done by the modifications of methods previously described (15). The cells were harvested by centrifugation at 4000 rpm for 30 min at 4°C and washed twice with 0.9% NaCl solution. The washed cells were suspended in buffer A (10 mM potassium phosphate, pH 7.2 containing 1 mM DTT and 0.1 mM EDTA) and disrupted by ultrasonic oscillator for 20 min. Cell debris were removed by centrifugation at 4000 rpm for 1 h at 4°C and the supernatant solution thus obtained, was used as the crude extract. The crude enzyme extract containing LeuDH was brought to 50% saturation by addition of solid ammonium sulfate while gently stirring, at 4°C. The precipitate was then collected by centrifugation at 4000 rpm for 60 min.

The pellet was redissolved in buffer B (buffer A containing 10% (v/v) glycerol) and dialyzed overnight at 4°C against at the same buffer. The dialyzed fraction was concentrated by ultrafiltration and subjected to a DEAE-Toyopearl column (diameter, 3 cm; length, 15 cm) equilibrated with buffer using a fast performance liquid chromatography (FPLC) system (Sykam, Germany). LeuDH was eluted with a stepwise linear salt gradient of NaCl concentration (50-150 mM) in buffer A at a flow rate of 2 ml/min. Fractions of 3 ml per tube were collected. The active enzyme fractions were pooled, concentrated, and stored at 4°C for various experiments. The concentrated enzyme was loaded on a TSK gel G3000SW column (0.7 cm × 60 cm), which had been equilibrated with buffer A. The flow rate was maintained at 0.5 ml/min. Fractions of 2 ml each was collected and absorbance at 280 nm was recorded.

### Determination of molecular weight of LeuDH

High performance liquid chromatography (HPLCsystem, Sykam, Germany) using TSK gel G3000SW was performed for the estimation of total molecular weight of LeuDH. A calibration curve was made with the following marker proteins: glutamate dehydrogenase (290,000), lactate dehydrogenase (142,000), enolase (67,000), and adenylate kinase (32,000). Purification process was analyzed by sodium dodecyl sulfate polyacrylamide gel electrophoresis (SDS-PAGE). SDS-PAGE was performed using discontinuous gels (10 cm × 10 cm) with a 6% stacking and a 12% separating gel. The protein samples were boiled for 5 min in 10 mMTris-HCl buffer (pH 7.0) containing 1% SDS, 80 mM 2-mercatoethonal and 15% glycerol. Electrophoresis was run at 30v and 10mA for 5h. Protein bands were visualized by staining with 0.025 Coomassie brilliant Blue R-250 in the mixture of 50% methanol and 10% acetate (16).

### Effect of temperature and pH on LeuDH activity

The effect of temperature on the enzymatic reaction of LeuDH was analyzed by assaying for the oxidative deamination activity at temperatures ranging from 40 to 80°C. Reaction mixture was pre-incubated at the desired temperatures for 4 min and the reaction was started by adding L-leucine followed by incubating for 4 min. The effect of pH on the enzymatic reaction of LeuDH was evaluated by measuring the oxidative deamination activity using the following buffer systems: 0.1 M sodium acetate (pH 3.0-5.0), 0.1 M potassium phosphate (pH 6.0-7.5), 0.1 M Tris-HCl (pH 8.0-9.0), 0.1 M glycine-NaOH (pH 9.0-11.0) and 0.1 M sodium carbonate (pH 11.5-12.0). All experiments were done triplicate and repeated at least twice (16).

### Coenzyme specificity and the effects of metal ions and chelating agent on the LeuDH activity

Coenzyme specificity in the oxidative domination and in the reductive animation assay was investigated. The inhibitory influences of metal ions and cheating agents were also measured (11).

### Kinetic studies

The kinetic parameters for the purified enzyme were calculated from the secondary plots of intercepts versus reciprocal concentrations of the other substrate (11).

## RESULTS

### Screening for thermophilic LeuDH enzyme

In order to isolate LeuDH producing thermophilic bacteria, 15 thermophilic bacterial strains were isolated from both soil and water samples from the Jask Port on a medium containing L-Leucine as the sole carbon and nitrogen source. Bacterial isolates, which were capable to utilize L-Leucine were cultivated in the production medium, and the enzyme activity was measured. Out of the 15 isolates, the isolate identified as JK-91 which exhibited L-Leucine (activity, 350 U/l) was selected for further studies.

### Characterization of strain JK-91

**Fig. 1:**
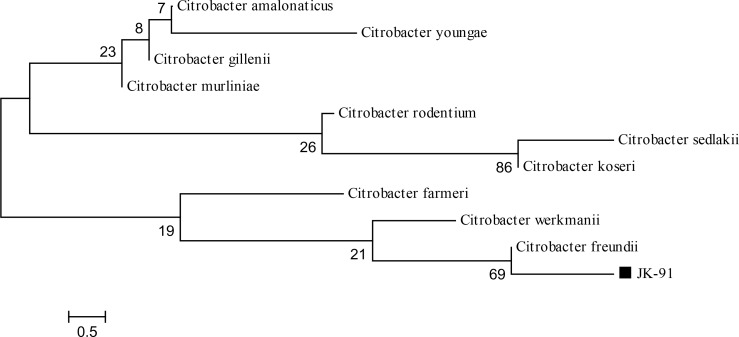
Phylogenetic tree based on 16r RNA sequences of the JK-91 isolate and closely related sequences. Numbers at nodes are levels of bootstrap support (%) based on Neighbor-joining (NJ) method of 1,000 resampled datasets. Isolate is marked.

The isolated bacterium was Gram negative, rod shape and motile. The results of the biochemical tests are shown in Table 1. Biochemical characterization revealed that isolate JK-91 might be similar to *Citrobacterfreundii*according the description in Bergey's manual of Determinative Bacteriology (13). Further identification was conducted by comparative sequence analysis of the 16S rDNA of isolate and other bacteria in the Genbank database. The 16S rDNA sequence of strain JK-91 had 80% similarity with the corresponding sequence of *C. freundii*. Multiple alignment and phylogentic analysis revealed the strain was closely related to *C. freundii*. According to the created phylogenetic tree based on 16S rDNA sequences (Fig. 1), the strain was recognized as *C. freundii* JK-91.

**Table 1:** Taxonomic characteristics of the JK-91 isolate.

Characteristic	JK-91
Shape	Rod
Colony morphology	Smooth, round, flat, glossy and 1-2 in diameter
Growth at Blood agar	Positive
Growth at MacConkey agar	Positive
Growth at 37°C	Positive
Gram stain	Negative
Triple SugarIron Agar (TSIA)	Alkaline/Acid
Methyl red (MR)	Positive
Citrate	Positive
Voges-Proskauer (VP)	Negative
Indole production	Negative
Urea hydrolysis	Negative
Oxidase reaction	Negative
Catalaze	Positive
H_2_S Production	Negative

### Biochemical properties of LeuDH

**Fig. 2:**
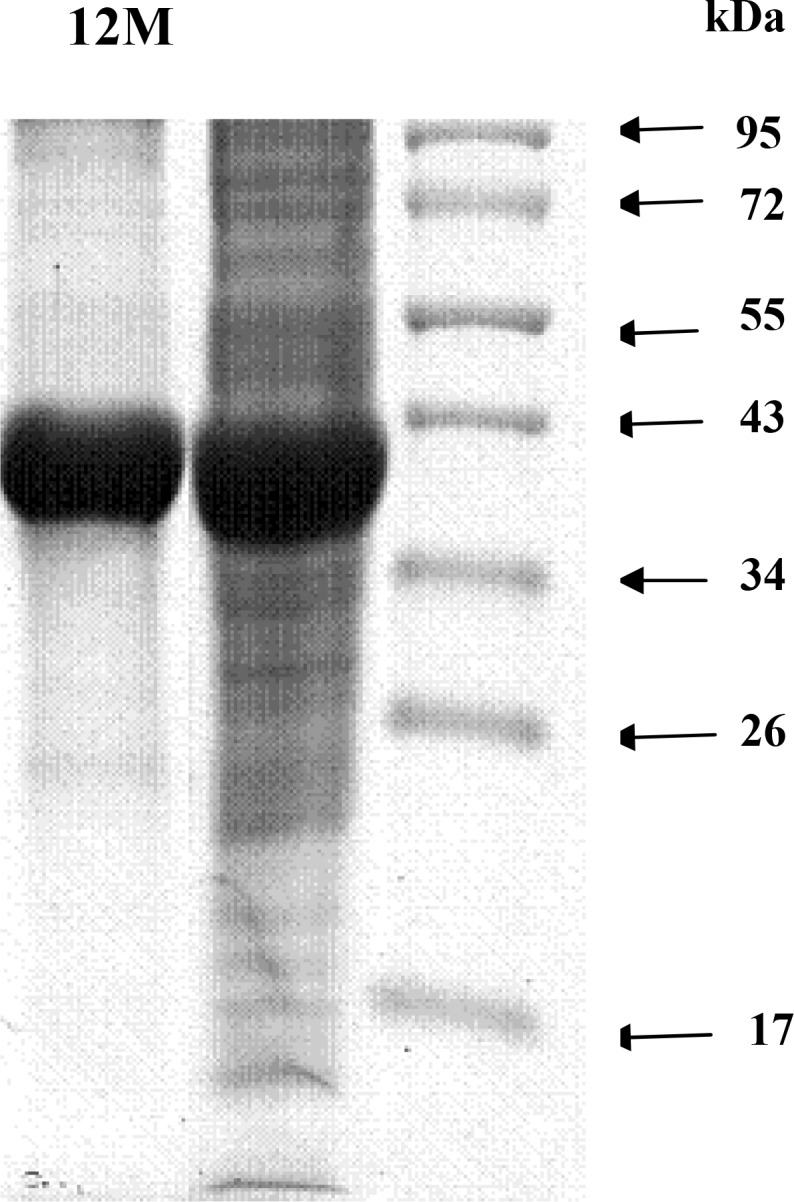
SDS-PAGE profile of purified *C. freundii* LeuDH. Lane 1: TSK gel G3000SW. Lane 2: crude extract. Lane M: molecular weight markers.

**Fig. 3:**
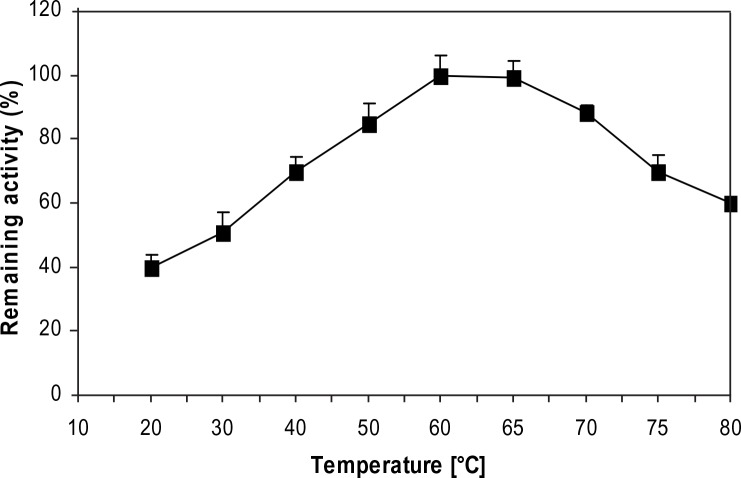
Influence of temperature on the activity of *C. freundii* LeuDH.

**Fig. 4:**
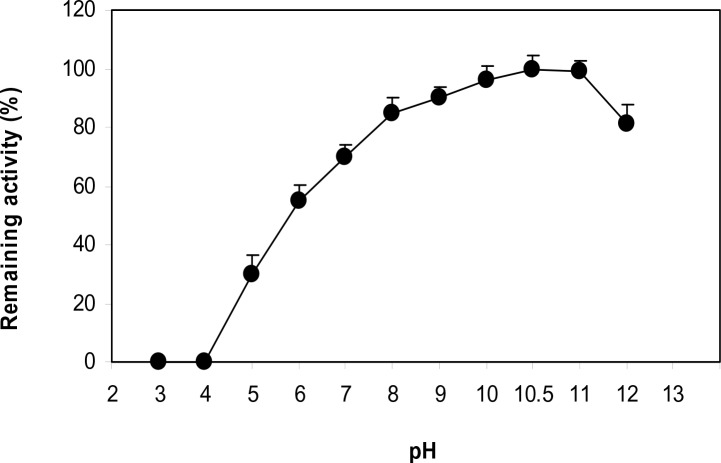
Influence of pH on the activity of *C. freundii* LeuDH.

The total molecular weight of LeuDH was determined as 320 Daltons by gel filtration method on TSK gel G3000SW. The molecular weight of the subunit was estimated to be 40 kDa by SDS-PAGE (Fig. 2). This finding showed that the enzyme consisted of eight subunits identical in molecular weight. The specific activity of the Purified LeuDH was 320 U/mg and comparable to LeuDH of other thermophilic bacteria. The substrate specificity of the LeuDH reaction with different substrates was examined. The following amino acids were inert for the LeuDH reaction: D-leucine, L-Arginine, Aspartate, and Glycine L-threonine. The enzyme required NAD and NADH as natural coenzymes, NADP and NADPH were inert. Chelating agents such as EDTA did not inhibit the enzyme. Meanwhile, metal ions such as Mg ^++^ and Ca^++^ did not inhibit or activate the enzyme activity. The *K*_m_ value of LeuDH reaction for leucine was also calculated to be 1.2 mM. The LeuDH retained its full activity of heating at temperature range of 50 to 75 °C, and its highest activity was achieved at 60 °C for 50 min (Fig. 3). The effect of various pH values on the enzymatic reaction of LeuDH was evaluated in the pH range from 3.0 to 12.0 at 70°C. LeuDH had a good activity in the range of pH 9.0-11.0 with optimal pH at 10.5 (Fig. 4).

## DISCUSSION

The search for extermophilic organisms is one of the means for finding enzymes with features suitable for industrial applications. The main advantage of thermostable enzymes is that they are able to tolerate higher temperature, which gives a longer half-life to the enzyme (1). Thermophilic enzymes not only tolerate high temperatures but conserve their activities under unfavorable conditions such as proteolysis, organic solutions, acidic and basic mediums and detergents (2, 7). LeuDH enzyme is recognized as a valuable biocatalyst for use in medical and pharmaceutical industries. A potential challenging area where LeuDH would have a critical role is the specific determination of blood L-leucine in relation to screen of MSUD. Regarding the LeuDH applications, finding enzyme with special features like temperature resistance or reaction in high saline concentrations is very interesting (1). Up to now, LeuDH has been isolated and characterized from different thermophilicstrains including *B. sphaericus, Thermoactinomyces intemediuc*, and *Clostridium thermoaceticus* (10, 17). In Iran, as said before, such a study to screen thermophilic LeuDHs has never been performed. Inthis study, we aimed to isolate a thermophilic LeuDH enzyme from the taken water and soil samples from the Jask Port. In the course of screening for the enzyme in thermophilic bacteria, only isolate JK- was found to be capable to produce LeuDH and was selected for further studies. To identify the strain JK-91, biochemical analysis was performed. The isolate was identified as a strain of *C. freundii* JK-91.SDS-PAGE gel analysis of the purified target enzyme showed a single band with an estimated molecular mass of 40 kDa. LeuDH studied so far are hexamers or octamers and the reported molecular mass for each subunit of enzyme varies from 40 kDa to 45 kDa (7, 17, 18). Therefore, the molecular weight of *C. freundii* JK-91 enzyme was in agreement with the available observations for LeuDH enzymes. The *C. freundii* JK-91 LeuDH had *K*_m_ value of 1.2 mM for leucine. This value was in agreement with those reported for the enzyme from *Clostridium thermoaceticus* (18), but differed from similar enzymes for *B. sphaericus* (10). The optimum pH was about 10.5. This was in accordance with the data to other LeuDHs from other bacteria such as *B. sphaericus* (10). Additionally, LeuDH from isolate JK-9 shares structural as well as mechanistic similarities to other LeuDHs reflected in its requirement of identical cofactor and substrates. Meanwhile, the higher thermal stability of *C. freundii* LeuDH toward leucine (60°C for 50 min) suggested that this enzyme would be very attractive for use in industry since the biotechnological applications of enzymes often depend on their stability in unfavorable environments.

In conclusion, a thermostable LeuDH was purified and characterized from a thermophilic strain of *C. freundii* JK-91 isolated from the Jask port. Collectively, LeuDH from *C. freundii* JK-91 appears to be useful for application in medical and pharmaceutical industries.

## References

[1] Seah SYK (2007). Amino acid dehydrogenases, Industrial enzymes. J. Polaina and A.P. Maccabe, Springer.

[2] Brunhuber NMW, Blanchard JS (1994). The biochemistry and enzymology of amino acid dehydrogenases. Crit Rev Biochem Mol Biol.

[3] Arthur JL Cooper, Myra Conway, Susan M Hutson (2003). A continuous 96-well plate spectrophotometric assay for branched-chain amino acid aminotransferases. Anal Biochem.

[4] Wendel U, Gonzales J, Hummel W (2003). Neonatal screening for maple syrup urine disease by an enzyme-mediated colorimetric method. Clin Chim Acta.

[5] Heldt K, Schwahn B, Marquardt I, Grotzke M, Wendel U (2005). Diagnosis of MSUD by newborn screening allows early intervention without extraneous detoxification. Mol Genet Metabol.

[6] Sofia Quental, Laura Vilarinho, Esmeralda Martins, Elisa LeãoTeles, Esmeralda Rodrigues, Luísa Diogo (2010). Incidence of maple syrup urine disease in Portugal. Mol Genet Metabol.

[7] Nagata S (1995). Gene cloning, purification, and characterization of thermostable and halophilic leucine dehydrogenase from a halophilic thermophile, *Bacilluslicheniformis* TSN9. Appl Microbiol Biotechnol.

[8] Misono H, Sugihara K, Kuwamoto Y, Nagata S, Nagasaki S (1990). Leucine Dehydrogenase from *Corynebacterium pseudodiphtheriticum*: purification and characterization. Agric Biol Chem.

[9] Katoh R, Ngata S, Ozawa A, Ohshima T, Kamekura M, Misono H (2003). Purification and characterization of leucine dehydrogenase from an alkaliphilic halophile, *Natrono bacteriummagadii*MS-3. J Mol Cata B: Enzym.

[10] Katoh R, Nagata S, Misoni H (2003). Cloning and sequencing of the leucine dehydrogenase gene from Bacillus sphaericus IFO 3525 and importance of the C-terimianl region for the enzyme activity. J Mol Cata B: Enzym.

[11] Shahbaz Mohammadia H, Omidinia E, Sahebghadam Lotfi A, Saghiri R (2007). Preliminary report of NAD^+^-dependent amino acid dehydrogenases producing bacteria isolated from soil. Iranian Biomed J.

[12] Baron EJ, Finegold SM (1990). Bailey and Scotts's diagnostic microbiology.

[13] Krieg NR, Holt JG (1984). Bergey's Manual of systematic Bacteriology.

[14] Tamura K, Dudley J, Nei M, Kumar S (2007). MEGA4: Molecular Evolutionary Genetics Analysis (MEGA) software version 5.0. Molecular Biology and Evolution.

[15] Kärst U, Schütte H, Baydoun H, Tsai H (1989). Purification and characterization of leucine dehydrogenase from the *Bacillus caldolyticus*. J. General Microbiol.

[16] Sambrook J, Fritsch E.F, Maniatis T (1994). Molecular Cloning: A laboratory manual.

[17] Nagata S, Bakthavatsalam S, Galkin AG, Asada H, Sakai S, Esaki N, Soda K, Ohshima T, Nagasaki S, Misono H (1995). Gene cloning, purification and characterization of thermostable and halophilicleucine dehydrogenase from a halophilic thermophile *Bacillus licheniformis* TSN9. Appl Microbiol Biotechnol.

[18] Shimoi H, Nagata S, Esaki N, Tanaka H, Soda K (1987). Leucine dehydrogenase of a thermophile anaerobe, *Clostridium thermoaceticus*: gene cloning, purification and characterization. Agric Biol Chem.

